# Novel Functions of Feedback in Electrosensory Processing

**DOI:** 10.3389/fnint.2019.00052

**Published:** 2019-09-13

**Authors:** Volker Hofmann, Maurice J. Chacron

**Affiliations:** Department of Physiology, McGill University, Montreal, QC, Canada

**Keywords:** descending pathways, weakly electric fish, response synthesis, neural coding, electrolocation, electrocommunication

## Abstract

Environmental signals act as input and are processed across successive stages in the brain to generate a meaningful behavioral output. However, a ubiquitous observation is that descending feedback projections from more central to more peripheral brain areas vastly outnumber ascending feedforward projections. Such projections generally act to modify how sensory neurons respond to afferent signals. Recent studies in the electrosensory system of weakly electric fish have revealed novel functions for feedback pathways in that their transformation of the afferent input generates neural firing rate responses to sensory signals mediating perception and behavior. In this review, we focus on summarizing these novel and recently uncovered functions and put them into context by describing the more “classical” functions of feedback in the electrosensory system. We further highlight the parallels between the electrosensory system and other systems as well as outline interesting future directions.

## Introduction

How sensory information is processed by the brain to give rise to behavior remains an important yet poorly understood question in systems neuroscience. This is due in part to the fact that, along a given sensory pathway, descending connections from higher brain areas (“feedback”) vastly outnumber ascending connections from the periphery (“feedforward”; Cajal, 1911; Holländer, 1970; Perkel et al., 1986; Sherman and Guillery, 2002; Markov et al., 2014; Salin and Bullier, 2017) and modify how sensory neurons respond to feedforward input. Previous studies have revealed multiple functions for such feedback pathways such as gain control (Treue and Martínez Trujillo, 1999), enhancing neural responses to particular stimuli (Hupé et al., 1998), or predictive coding (Bastos et al., 2012). Recent research in the electrosensory system of weakly electric fish has revealed novel, qualitatively different functions for feedback pathways in that their transformations of feedforward signals can generate neural responses that mediate behavioral responses to sensory input. Here, we review these novel functions and provide context for these results, particularly with regards to other previously established functions of feedback in the electrosensory as well as in other systems.

## The Electric Sense: Relevant Neural Circuitry and Sensory Input

Weakly electric fish as a model system benefit from well-characterized anatomy, natural stimuli, as well as feedback circuits that are easily accessible for pharmacological manipulation. We note that all these features have been extensively reviewed elsewhere (Bastian, 1999; Berman and Maler, 1999; Bell and Maler, 2005; Chacron et al., 2011; Marsat et al., 2012; Krahe and Maler, 2014; Metzen et al., 2017).

**Figure 1:**
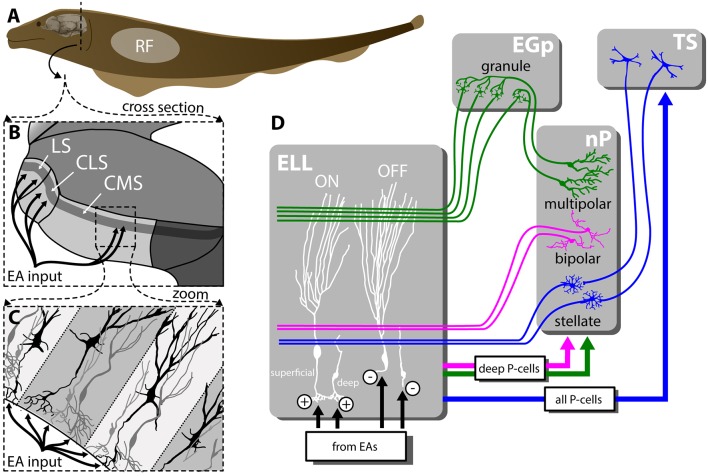
Feedforward and feedback connectivity of the ELL. **(A)** Electroreceptors distributed across the body surface encode electrosensory stimuli and project to pyramidal cells (P-cells) within the hindbrain ELL. **(B)** The ELL is organized in three parallel segments (LS, CLS and CMS), each of which is a somatotopic representation of the body surface. All segments receive the same feedforward input from EAs. **(C)** Pyramidal cells (P-cells) within all ELL segments are organized in columns. P-cells receive feedforward input at their basal sites whereas feedback inputs project to the apical dendrites. **(D)** While ON-type P-cells receive direct excitatory (“+”) input from peripheral electroreceptors, OFF-type P-cells instead receive indirect inhibitory input *via* local interneurons (“−”). While all P-cells project to the midbrain TS, only a subset of P-cells whose somata are located deep within the pyramidal cell layer (i.e., “deep” P-cells) project to the nP. There are several sources of feedback onto ELL P-cells: one of the pathways forms a closed-loop and is topographically ordered. It consists of ascending projections from all P-cells to TS from where descending projections project onto stellate cells within nP that then project back to ELL P-cells with direct excitation and indirect inhibition *via* local interneurons (blue). The second pathway consists of feedforward projections from deep P-cells to bipolar cells within nP that then project back to ELL P-cells in a diffuse manner and in an inhibitory fashion (magenta). The third pathway is termed “indirect” and consists of feedforward projections from deep P-cells to multipolar cells within nP that then project to granule cells within the EGP which make parallel fiber connections to ELL P-cells. It should be noted that such parallel fibers make little if any synaptic contact with deep P-cells. As such, this indirect pathway forms an open loop. Abbreviations: EA, electrosensory afferents; EGP, eminentia granularis posterior; ELL, electrosensory lateral line lobe; CLS, centrolateral segment; CMS, centromedial segment; LS, lateral segments; nP, nucleus praeminentialis; RF, receptive field; TS, torus semicircularis.

To sense their surroundings and communicate with conspecifics, Gymnotiform weakly electric fish actively generate an electric field by emitting a quasi-sinusoidal electric organ discharge (EOD). Objects with a conductivity different from that of the surrounding water (e.g., prey) as well as interactions with the EOD of a conspecific will modulate the amplitude of the animal’s EOD. These changes in amplitude are detected by electroreceptors scattered on the animal’s skin (Scheich et al., 1973) that synapse onto pyramidal cells (P-cells) within the electrosensory lateral line lobe (ELL; Maler, 1979; Maler et al., 1981; Figure 1A). Specifically, each afferent fiber trifurcates to make synaptic contact with P-cells within three parallel segments: the centromedial (CMS), centrolateral (CLS) and the lateral (LS) segments (Figure 1B) that are organized in columns (Figure 1C) and display large differences in terms of receptive field organization (Shumway, 1989; Krahe et al., 2008; Hofmann and Chacron, 2017), ion channel composition (Ellis et al., 2008; Motipally et al., 2019), and responses to electrosensory stimuli (for review, see Krahe and Maler, 2014). There are two main types of P-cells: ON- and OFF-type that respond to increases and decreases in EOD amplitude, respectively (Maler, 1979; Maler et al., 1981; Saunders and Bastian, 1984). All P-cells project directly to the midbrain Torus semicircularis (TS) and, from there, indirectly to higher brain areas. There are large morphological and functional heterogeneities in the P-cell population (Maler, 2009a,b). In particular, so-called “deep” P-cells display small apical dendrites and receive little feedback, whereas “superficial” P-cells instead display large apical dendrites and receive large amounts of feedback.

P-cells receive large amounts of feedback from higher brain centers (Sas and Maler, 1983, 1987) that consist of three major pathways. Neurons within TS project back to stellate cells within the nucleus praeminentialis (nP) that then in turn project back to ELL P-cells with direct excitation and indirect inhibition through local interneurons (Figure 1D, blue). This feedback pathway forms a closed loop and is part of the “direct feedback pathway” (Bratton and Bastian, 1990; Berman and Maler, 1999). Bipolar and multipolar cells, as well as other cell types within nP, receive input from deep P-cells (i.e., only a sub-set of ELL output). While bipolar cells project back to ELL pyramidal cells in an inhibitory fashion forming the other part of the “direct pathway” (Figure 1D, magenta), multipolar cells instead project indirectly to ELL *via* granule cells of the eminentia granularis posterior (EGp; Bastian and Bratton, 1990). The ELL is a cerebellar-like structure, as EGp granule cell axons form parallel fibers that contact the apical dendrites of superficial P-cells (Bastian et al., 2004; Figure 1D, green). This open-loop feedback is known as the “indirect pathway.” It should be noted that ELL pyramidal cells also receive other sources of neuromodulatory feedback (e.g., serotonergic, cholinergic; for review, see Márquez et al., 2013).

Electrosensory stimuli comprise of EOD amplitude modulations caused by prey or inanimate objects which are spatially localized (i.e., they impinge only upon a fraction of the animal’s skin surface; Nelson and MacIver, 1999; Pedraja et al., 2018) or those caused by interactions with conspecifics that are spatially diffuse (i.e., they impinge on most if not all the animal’s skin surface). In the latter case, interactions between the EODs of two conspecifics give rise to a sinusoidal amplitude modulation (i.e., a beat) whose frequency depends on the difference between the two individual EOD frequencies. The beat amplitude, termed the envelope, depends on the relative distance and orientation between both fish (Yu et al., 2012; Fotowat et al., 2013).

## Functions of Feedback Input Onto ELL Pyramidal Cells

In this section, we will briefly summarize some of the established functions of feedback pathways on electrosensory processing towards modifying how P-cells respond to feedforward input from the periphery (e.g., response enhancement or attenuation). In the following section (see “Recently Uncovered Novel Functions for Electrosensory Feedback” section), we will then focus on recently uncovered novel functions of electrosensory feedback, that is the generation of neural responses which mediate perception and behavior.

### Gain Control and Adaptive Cancelation of Sensory Stimuli

The first reported *in vivo* manipulations of electrosensory feedback pathways consisted of pharmacological inactivation as well as of lesioning the indirect feedback pathway (Bastian, 1986a,b). These manipulations made pyramidal cells more sensitive to changes in beat amplitude (i.e., increased the gain, which constitutes divisive gain control). This phenomenon has been studied in great theoretical detail (Lewis and Maler, 2004; Mejias et al., 2014) and a careful analysis revealed that there appears to be both divisive and subtractive (i.e., a shift in the sensitivity curve) gain control features.

Further studies have shown that the indirect pathway cancels predictable (i.e., redundant) sensory input *via* the formation of a “negative image” whose amplitude can be modulated to match that of the feedforward sensory input through plasticity at feedback synapses (for review, see Bastian, 1999). Moreover, investigators have focused on how recently-uncovered synaptic plasticity rules mediate the formation of the negative image (Bol et al., 2011; Harvey-Girard and Maler, 2013), thereby making neural responses more invariant with respect to stimulus amplitude (Mejias et al., 2013). The indirect feedback pathway is diffuse in nature and is primarily activated by spatially diffuse but not by spatially localized stimuli (Chacron et al., 2003, 2005b; Bastian et al., 2004; Chacron, 2006). It most likely originates from the so-called “non-classical” receptive field (i.e., the area of sensory space in which impinging stimuli do not by themselves affect the neural response but can modulate the response to stimuli impinging upon other areas of sensory space). Through this feedback input ELL pyramidal cells responses to low frequency stimuli are attenuated (Chacron et al., 2003, 2005a) and the responsiveness to spatially localized (e.g., prey) stimuli is increased (Litwin-Kumar et al., 2012). Moreover, as the indirect feedback pathway is primarily activated by low-frequency stimuli (Chacron et al., 2005b), another function is the enhancement of responses to high-frequency electrocommunication stimuli (Chacron et al., 2003, 2005b; Bastian et al., 2004; Chacron, 2006; Marsat and Maler, 2012; Marsat et al., 2012; Metzen, 2019) and the cancelation of re-afferent sensory input that is self-generated during tail motion (Bastian, 1995, 1996; Lewis et al., 2007). Most recently, it was shown that the indirect pathway is also involved in attenuating responses to low frequency envelopes (Huang et al., 2018), which is consistent with the above-mentioned fact that feedback makes neural responses to first-order stimuli more invariant with respect to stimulus amplitude (Mejias et al., 2013). It should be emphasized here that the indirect feedback pathway forms an open loop in that it originates from deep P-cells and primarily terminates on superficial P-cells. Modeling studies suggest that such a configuration is necessary for adaptive cancelation to occur (Bastian et al., 2004).

Similar functions have also been uncovered in other species of weakly electric fish (Enikolopov et al., 2018) and also show striking parallels to both the auditory and the visual system: the enhancement of responses to high-frequency stimuli through the attenuation of responses to low frequency stimuli is similar to what is seen in the auditory system. Here, feedback signals from the corticofugal system modulate frequency tuning of auditory subcortical neurons *via* synaptic plasticity (Chowdhury and Suga, 2000; Gao and Suga, 2000; Ma and Suga, 2001). Also, feedback was shown to effectively cancel responses to self-generated sounds in the dorsal cochlear nucleus (Singla et al., 2017). The fact that electrosensory feedback originates from a “non-classical” part of the receptive field and modulates responses of target neurons (see above), is reminiscent to the visual system. Their stimulation outside of the receptive field was shown to effectively enhance the responses of neurons within the primary visual cortex to visual edges (i.e., high-spatial frequency stimulus features) in natural visual scenes (Vinje and Gallant, 2000, 2002), presumably through feedback signals. Similarly, stimulation of feedback to thalamic neurons was shown to effectively enhance their classical surround, thereby increasing sensitivity to high-spatial frequency stimuli (Murphy and Sillito, 1987; Sillito et al., 1993; Cudeiro and Sillito, 1996; Jones et al., 2000; Webb et al., 2003).

### Generation of Gamma-Range Oscillations

Oscillatory neural activity within the gamma band (20–80 Hz) is seen ubiquitously across systems and species and is thought to play an important role in information processing (Uhlhaas et al., 2011; Buzsáki and Wang, 2012). During diffuse stimulation, inhibitory input from nP bipolar cells (Figure 1D, magenta) was shown to generate a gamma oscillation which can be seen in the activities of single ELL pyramidal cells (Doiron et al., 2003). Specifically, the transmission delay associated with this feedback pathway (~15 ms) gives rise to a peak in spectral power (~30 Hz). No such oscillations were seen when spatially localized stimuli mimicking prey were used instead. Subsequent studies have shown that the induced oscillations not only require spatially diffuse stimulation but also that the stimuli need to be spatially correlated with one another (Doiron et al., 2004; Lindner et al., 2005).

Further theoretical studies have highlighted potential issues with the original modeling as it did not specifically account for the fact that there are both ON- and OFF-type ELL pyramidal cells that respond to increases and decreases in the stimulus, respectively (Lefebvre et al., 2009; Payeur et al., 2013). If the feedback pathways simply integrate input from both cell types, then the power of gamma band oscillations would be weak, which is unlike what is observed experimentally. Based on these studies, several predictions regarding the anatomical organization of the feedback pathway were made: the feedback should be strongly asymmetric or segregated between ON- and Off-type responses. An oscillation resulting from stellate cell feedback seems however unlikely given that blocking excitatory feedback from nP stellate cells *in vivo* did not alter this oscillation (Doiron et al., 2003). Another alternative is that these oscillations result from a combination of feedforward excitatory and delayed feedforward inhibitory inputs that can mimic weak oscillatory states (Payeur et al., 2015).

Functionally, gamma-oscillations might enable the animal to distinguish between prey and conspecific-related stimuli (Doiron et al., 2003), or to enhance the ability of TS neurons to encode motion direction (Ramcharitar et al., 2005). Such functions are similar to those found in mammalian systems, where cortico-thalamic feedback loops generate multiple rhythms that drive neocortical neurons to fire in synchrony and thus presumably better encode specific stimulus features (for review, see Nuñez and Malmierca, 2007). Whether gamma-range oscillations synchronize the ELL pyramidal cell network remains to be shown experimentally. While only a limited number of studies has recorded from ELL pyramidal cell pairs so far (Chacron and Bastian, 2008; Litwin-Kumar et al., 2012; Simmonds and Chacron, 2015; Hofmann and Chacron, 2018b), answering the above question will require recordings from even greater population sizes. Such studies should also investigate if and how such synchronization enables the ELL pyramidal cell network to better encode behaviorally relevant stimuli.

### Sensory Searchlight

While clear functional roles for both the indirect feedback as well as the direct feedback (bipolar component) were uncovered, the functional role of direct feedback pathway emanating from nP stellate cells has remained elusive. This is despite the fact that multiple studies have characterized how stellate cells respond to relevant electrosensory stimuli *in vivo* (Bratton and Bastian, 1990), characterized synaptic plasticity at stellate to P-cell synapses both *in vitro* (Oswald et al., 2002) and *in vivo* (for review, see Bastian, 1999). Because this pathway is topographic in nature, it was hypothesized that it should be primarily activated by spatially localized stimuli. Moreover, because of the strong potentiation observed at synapses, it was thought that this pathway acts as a “sensory searchlight” by enhancing P-cell responses to spatially localized stimuli (Berman and Maler, 1999). While it is true that stellate cells respond to stimuli mimicking prey (Bratton and Bastian, 1990), there has been, at least until very recently, no direct demonstration of a function for the direct feedback pathway *in vivo*. We next describe recently uncovered functions for this pathway.

## Recently Uncovered Novel Functions for Electrosensory Feedback

### Generation of Bursting Neuronal Responses to Moving Objects

A recent study has investigated how electrosensory feedback pathways affect P-cell responses to moving objects (Clarke and Maler, 2017). While previous studies have investigated how electrosensory neurons respond to objects moving along the animal’s rostro-caudal axis (Bastian, 1981a,b; Saunders and Bastian, 1984; Chacron et al., 2009; Khosravi-Hashemi et al., 2011). Clarke and Maler (2017) have instead investigated how electrosensory neurons responded to looming and receding objects. Such stimuli are experienced by the animal during the electromotor response (Heiligenberg, 1973; i.e., when animals seek to maintain a constant lateral position to large moving objects such as root masses of plants). Their stimulation paradigm consisted of a looming object that would then remain stationary close to the animal’s skin surface. After a few seconds, the object was again receded from the skin surface. The authors used both metal as well as plastic objects that will increase and decrease EOD amplitude, respectively. They found that peripheral electroreceptors displayed strong adaptation to both looming and receding objects (i.e., their firing rates returned to values seen in the absence of stimulation; Clarke et al., 2013). Interestingly and unlike afferents, an increase in firing rate due to burst firing was also observed when an “inverted-contrast” paradigm was used for receding motion (i.e., OFF-type cells with a metal object or ON-type cells with a plastic object). This burst response was seen even after the object remained stationary close to the animal’s skin for several seconds during which the firing rate of peripheral electroreceptors almost fully adapted (Clarke et al., 2014).

How can P-cells give such a strong burst response even though the peripheral electroreceptors that provide feedforward input do not? Clarke and Maler (2017) investigated the role of ELL feedback pathways towards generating responses to both looming and receding objects. To do so, they blocked descending input from TS onto nP stellate cells (Figure 1D, blue). While such a manipulation only moderately affected responses to looming objects, they found that burst responses to receding objects were abolished. How can closed-loop feedback from TS to nP stellate cells to ELL generate bursting responses in P-cells to receding objects? P-cells display a burst mechanism that relies on somato-dendritic interactions (Lemon and Turner, 2000; Krahe and Gabbiani, 2004; Metzen et al., 2016). Generally burst firing serves to signal specific stimulus features (Oswald et al., 2004; Maler, 2009a; Avila-Akerberg et al., 2010; for review, see Krahe and Gabbiani, 2004). Here, the authors propose that feedback input from nP stellate cells is far more effective at eliciting burst firing from P-cells because these synapses display strong potentiation (Oswald et al., 2002). In contrast, the feedforward input from peripheral electroreceptors will be too weak to elicit a burst response by itself. Thus, this mechanism requires feedforward input to elicit feedback that then generates the neural burst response through a transformation of the feedforward input. Specifically, electroreceptor responses elicit P-Cell isolated spikes (i.e., no bursts) which then trigger bursting *via* feedback. Interestingly, further studies have shown that serotonergic modulation can enhance P-cells burst responses to improve the detectability of receding but not looming objects (Marquez and Chacron, 2018). Further experiments are needed to fully understand the mechanisms by which feedback pathways generate responses to moving objects.

### Generating Neural Responses to Envelopes

In a series of experiments, Metzen et al. (2018) have found that, for low enough beat amplitudes (i.e., envelopes), feedback pathways are necessary to generate both neural and behavioral responses. Such stimuli would occur when both animals are located far away from each other (Stamper et al., 2013). The authors used sinusoidal beat stimuli whose amplitude (i.e., envelope) increased linearly over time and measured both neural and behavioral detection thresholds (i.e., the stimulus amplitude for which the neural or behavioral response became significantly different from that seen in the absence of stimulation). Responses were quantified by either the mean firing rate or the strength of phase locking to the beat.

Behaviorally, animals respond to very faint envelope stimuli (i.e., <10% contrast) through modulation of their EOD frequency. When investigating the neural underpinnings of these behavioral responses, it was found that, although ELL P-cells could detect faint envelope stimuli through increases in firing rate, peripheral electroreceptors did not. Their firing rates only showed significant increases at much higher (>30%) stimulus amplitudes. This finding is almost paradoxical: how can ELL P-cells respond through changes in firing rate to stimuli, even though their input does not? The answer lies beyond firing rate: if one considers detection thresholds based on phase locking, then both peripheral electroreceptors and P-cells actually respond to faint envelope signals (i.e., contrasts of less than 10%). Thus, a simple explanation for the observed behavioral responses is that information transmitted in a feedforward fashion *via* phase locking elicits behavioral responses. However, when eliminating feedback from nP stellate cells *via* injection of the sodium channel antagonist lidocaine, this explanation was proven wrong: both behavioral and P-cell firing rate responses to faint envelope signals were abolished. Interestingly, feedback manipulation did not affect phase locking in P-cells and as such, it is the P-cell firing rate response rather than phase locking that is decoded in order to give rise to behavior.

Other control experiments showed that blocking the indirect pathway did not affect P-cell responses and that injecting lidocaine into TS gave rise to the same effects on P-cell responses as those obtained after injecting lidocaine in nP. Recordings from stellate cells in nP showed an increase in firing rate to faint envelope signals. Both of these controls strongly suggest that the phase locking responses of P-cells are inherited from those of their afferent (i.e., feedforward) inputs and that these are transformed into a firing-rate response within the closed-loop feedback of the direct topographic pathway. A further study has shown that this pathway increases P-cell firing rate responses to envelopes independently of temporal frequency (Huang et al., 2018). Thus, phase locking from peripheral receptors induces phase locking in P-cells that presumably triggers an increase in the firing rate of nP stellate cells, which in turn increases P-cell firing rate response which is decoded downstream to give rise to behavior. Further studies are however needed to fully understand the mechanism that transforms phase locking to firing rate and generates P-cell firing rates responses to faint envelope signals. It should be noted that, in this case, ELL pyramidal cell phase locking responses (i.e., temporal code) are preserved and used to generate firing rate responses (i.e., a rate code). This shares similarities with the somatosensory system of rodents where cortical feedback transforms an incoming temporal code into a rate code (Ahissar et al., 2000).

## Summary and Future Directions

In this review, we have summarized the classical functions of electrosensory feedback to highlight recently uncovered novel functions of the closed-loop direct pathway towards generating neural and behavioral responses. The functions of feedback in the ELL are summarized in Table 1.

**Table 1:** Functions of feedback in the electrosensory system.

	“classical” functions			
Function	Pathway	Type	Effective transformation	Studies
Cancelation of redundant LF input	Indirect, diffuse	Open loop	Generation and scaling of a negative image	Bastian (1995, 1999) and Bastian et al. (2004)
Control of frequency tuning	Indirect, diffuse	Open loop	Generation and scaling of a negative image for low frequency stimuli.	Chacron et al. (2003, 2005b), Chacron (2006); Huang et al. (2016, 2018) and Huang and Chacron (2017)
Induction of oscillation in gamma range	Direct, diffuse	Open loop	Delayed inhibitory feedback, interaction with STSP of ELL efferents	Doiron et al. (2003, 2004) and Lindner et al. (2005)
Sensory searchlight (?)	Direct, topographic	Closed loop	Excitatory input triggering burst firing	Berman and Maler (1999)
	**“novel” functions**			
Generation of responses to receding objects	Direct, topographic	Closed loop	Excitatory input triggering burst firing	Clarke and Maler (2017)
Generation of envelope responses at low contrasts	Direct, topographic	Closed loop	Transformation of phase locking to firing rate.	Huang et al. (2018) and Metzen et al. (2018)

It is likely that similar roles of feedback can be found in other systems. This is because the electrosensory system shares many similarities with both the visual (for review, see Clarke et al., 2015) as well as auditory and vestibular systems (Metzen et al., 2015). On top of examples mentioned above, recent studies have shown that feedback is necessary to complete the perception of touch (Manita et al., 2015; Kwon et al., 2016; Takahashi et al., 2016). Specifically, such feedback terminates on the apical dendrites of cortical neurons to generate a burst response, which is conceptually similar to the results of Clarke and Maler (2017). In neurons of the cochlear nucleus, detection thresholds to envelopes are thought to emerge through feedforward integration of input from auditory fibers. However, based on the results of Metzen et al. (2018), further studies should investigate how feedback contributes to determining auditory envelope detection thresholds.

In the electrosensory system, a better understanding of the mechanisms underlying the described feedback transformations will only be achieved by investigating how TS neurons respond to electrosensory stimuli (Khosravi-Hashemi et al., 2011; Vonderschen and Chacron, 2011; McGillivray et al., 2012; Sproule et al., 2015). Also, further studies will require the characterization of responses in nP using stimuli like those in Clarke and Maler (2017) and Metzen et al. (2018). In addition to that, further studies are needed to understand the interplay between neuromodulatory feedback and the feedback inputs described here and how this optimizes neural responses based on behavioral context. Beyond that, it is clear that, in the electrosensory as in other systems, behavior is determined by integrating the activities of large neural populations. However, only a few studies have begun to unravel the impact of feedback on population coding (Chacron and Bastian, 2008; Litwin-Kumar et al., 2012; Simmonds and Chacron, 2015; Hofmann and Chacron, 2018b). This research direction is particularly interesting and timely as feedback has been shown to impact population coding in other systems (Bondy et al., 2018; Merrikhi et al., 2018).

Finally, it should be noted that most studies have investigated the impact of feedback onto neuronal coding in immobilized and thus, at least partly, behaviorally restrained animals. Nonetheless, animals in general and weakly electric fish in particular are known to display a rich repertoire of sensory-related behaviors termed “active sensing movements” (for review, see e.g., Schroeder et al., 2010; Wachowiak, 2011; Hofmann et al., 2013; Grant et al., 2014). It was shown that active control of the re-afferent sensory input can enhance (Stamper et al., 2012; Hofmann et al., 2017) or even generate sensory information (Biswas et al., 2018; Hofmann and Chacron, 2018a; Pedraja et al., 2018). Recent technological advances made it possible to record neuronal activity in freely moving animals (Fotowat et al., 2019) and thus to investigate the role of feedback under active conditions. Such studies focus on how feedback is involved in differentiating between ex- and re-afferent input, and in shaping neuronal tuning in an activity-based manner. It is likely that such mechanisms are similar to those encountered in the somatosensory system of whisking rodents, where cortical feedback unto thalamic neurons can, for example, alter receptive field size thereby enhancing sensory information (Malmierca and Nuñez, 2004).

## Author Contributions

VH and MC conceptualized the review and revised the manuscript to its final version. VH prepared the figure, table and wrote the initial draft.

## Conflict of Interest Statement

The authors declare that the research was conducted in the absence of any commercial or financial relationships that could be construed as a potential conflict of interest.
